# Endotoxins and Metabolic Endotoxemia in Obesity and Associated Noncommunicable Diseases: A Focus on Sex Differences

**DOI:** 10.3390/biom16020226

**Published:** 2026-02-02

**Authors:** Manuela Del Cornò, Anna Aureli, Barbara Varano, Lucia Conti

**Affiliations:** 1 Center for Gender-Specific Medicine, Istituto Superiore Di Sanità, 00161 Rome, Italy; barbara.varano@iss.it; 2 Institute of Translational Pharmacology, National Research Council, 67100 L’Aquila, Italy; anna.aureli@cnr.it

**Keywords:** obesity, endotoxin, sex differences, metabolic endotoxemia, inflammation, diet, noncommunicable diseases

## Abstract

Metabolic endotoxemia has been proposed as a possible mechanism to explain the strong link between inflammation, obesity, and obesity-associated disorders. Gut dysbiosis is a hallmark of obesity, and diet has been reported to regulate both inflammation and disease risk by affecting the composition of gut microbiota and gut barrier function. In the condition of microbial imbalance and impaired intestinal mucosa, bacterial endotoxins, specifically lipopolysaccharides, translocate from the gut into the bloodstream, where they can sustain a prolonged, sterile, low-grade inflammation, raising the risk of several non-communicable diseases. Increasing evidence indicates that the risk and incidence of obesity and several obesity-associated disorders are sex-specific, although the underlying mechanisms are only just emerging. Notably, most of the factors influencing metabolic endotoxemia exhibit sexual dimorphism. This review aims to summarize the human studies investigating the role of metabolic endotoxemia in obesity and associated diseases, with a focus on those highlighting sex differences. We also discuss the clinical relevance of circulating endotoxins in metabolic derangements and their potential role as sex-related and modifiable risk factors to consider in future prevention strategies.

## 1. Introduction

**Figure 1:**
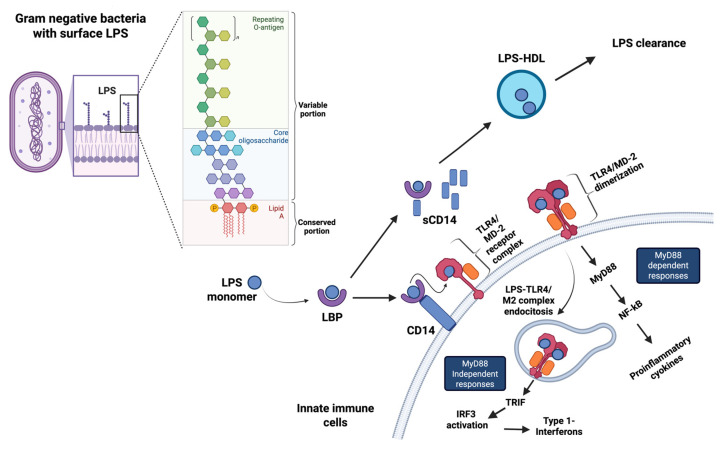
Schematic overview of the structure and the mechanism of action of LPS on innate immune cells. (**Left**): The three main regions of bacterial LPS are depicted. LPS monomers are released from bacterial membranes and then transferred by the serum LPS binding protein (LBP) to membrane-bound or soluble CD14 molecule (sCD14). (**Top right**): LBP and sCD14 act as a shuttle to move LPS to high-density lipoproteins (HDL), which neutralize its inflammatory effects. (**Bottom right**): In dysbiosis, high levels of LBP-LPS complex delivers LPS to mCD14 that transfers the LPS to the myeloid differentiation factor 2/Toll-like receptor 4 (MD-2/TLR4) complex on the innate immune cells. Dimerization of TLR4 then initiates two main downstream intracellular signaling pathways: the MyD88-dependent (NF-κB pro-inflammatory cytokines response) and the MyD88-independent (TRIF-IRF3-IFN I axis). Created in BioRender. Anna Aureli. (2026) https://BioRender.com/ (accessed 14 January 2026).

Endotoxins or lipopolysaccharides (LPS) are large molecules found in the outer membrane of Gram-negative bacteria and endowed with a potent immunostimulatory activity [1]. They are composed of a hydrophobic lipid A region embedded in the bacterial membrane, responsible for receptor recognition and immunoregulation, a hydrophilic core oligosaccharide, and a repeating O-antigen polysaccharide side chain that extends outwards, conferring antigenic specificity [2] (Figure 1). LPS has been widely studied in the context of acute bacterial infections and in severe sepsis [3]. However, growing evidence indicates that persistent low levels of endogenous endotoxins, released by the microbiota, can sustain prolonged sterile inflammation or meta-inflammation, which is a typical feature of obesity and contributes to increasing the risk of several noncommunicable diseases (NCD) [4].

Overweight and obesity—whose prevalence has risen to epidemic proportions over the past 50 years and is forecasted to further increase—remain one of the most common public health issues in the developed world. Obesity is a complex and heterogeneous disease, characterized by a chronic state of oxidative imbalance and inflammation at both adipose tissue (AT) and systemic level, and represents the main risk factor for several metabolic disorders, cardiovascular diseases (CVD), neurodegenerative diseases (ND), and cancers [5]. Worldwide, women have a higher likelihood of becoming obese compared to men, whereas men seem to be more susceptible to the metabolic complications of obesity [6]. Likewise, the incidence and onset of several obesity-associated disorders show sex disparity, although the study of sex differences is still neglected, and the underlying mechanisms are only just emerging.

Gut dysbiosis is a hallmark of obesity and results from a loss of bacterial diversity and altered microbial composition, often characterized by a contraction of the highly abundant Firmicutes and Bacteroidetes phyla and parallel outgrowth of pro-inflammatory phyla, including Gram-negative Proteobacteria, which are the main source of endotoxins [7]. Additionally, in individuals with obesity, the intestinal microbiota exhibits a decrease in the abundance of Bifidobacteria and Lactobacillus, which are beneficial for maintaining the integrity of the intestinal barrier. This imbalance disrupts normal functions like nutrient absorption and immune regulation, and increases the release of bacterial metabolites, antigens, and toxins.

The resulting gut barrier permeability or leaky gut acts as a driver for luminal bacteria and bacterial endotoxins to enter the bloodstream, leading to metabolic endotoxemia and contributing to chronic subclinical systemic inflammation. Correct lifestyle, and particularly healthy diets, have been reported to restrain inflammation and to reduce the risk of obesity and related disorders by maintaining gut barrier function and homeostasis [7]. Alteration of the oral microbiota has also been increasingly implicated in obesity and in the pathogenesis of chronic diseases. The concept of an oral-blood axis has been proposed, indicating the potential for oral bacteria and endotoxins to enter the bloodstream, further propagating inflammation and metabolic dysfunction [8].

LPS and other microbial products are released in the luminal compartment following bacterial lysis, dissociated during microorganism growth, or included in outer membrane vesicles released by live bacteria [1]. They translocate from the gut lumen into the blood at low levels through epithelial cell tight junctions in a natural and controlled process [1]. However, in the condition of dysbiosis, when intestinal permeability increases due to a high-fat diet (HFD), inflammation, oxidative stress, and blood translocation of higher levels of endotoxins occur, and pathogenic events are triggered [9,10]. A two- to three-fold increase in plasma LPS concentration (1–10 ng/mL), which, however, remains 10–50 times lower than those achieved in sepsis, characterizes metabolic endotoxemia [11].

As shown in Figure 1, once in the circulation, LPS binds to lipopolysaccharide-binding protein (LBP), which delivers it to the CD14/Toll-like receptor 4 (TLR4) receptor complex expressed on innate immunity cells and rapidly triggers pro-inflammatory signaling cascades [12]. Conversely, the soluble CD14 (sCD14) isoform, generated during sustained endotoxin exposure, mediates a protective role by shuttling LPS to high-density lipoproteins (HDL) for hepatic clearance [2,12]. The LBP-sCD14-HDL axis plays a critical regulatory role in detoxifying and removing LPS from the circulation, thereby preventing excessive immune activation [2,10]. For their longer half-life in the blood compared to LPS, LBP and LBP/sCD14 ratio are recognized as the main clinical markers of metabolic endotoxemia [10]. When the equilibrium is disrupted, as indicated by a high plasma LBP:sCD14 ratio, LPS engages TLR4 on both immune and non-immune cells, and triggers signaling cascades leading to the chronic release of pro-inflammatory and pro-oxidant mediators [12].

This chronic activation of the immune system finally results in immunosuppression that plays a central role in the pathogenesis of obesity, insulin resistance (IR) and related chronic diseases [13,14,15]. High LPS concentrations have been measured in the blood of subjects with obesity, type 2 diabetes mellitus (T2DM), CVD, ND and cancers. Moreover, LPS can be detected in the circulation of healthy individuals with levels transiently increasing following consumption of fat-enriched foods [16]. The biological consequences of endotoxemia, however, are critically influenced by the type and structural characteristics of LPS. The pro-inflammatory potential, or endotoxicity, of lipid A varies among Gram-negative bacterial species, according to its structure and acylation/phosphorylation degree that influence receptor binding, and endotoxins from some commensal species can exert lower or even opposite effects on host immune/inflammatory response [17,18]. This underlines that the relative abundance of microbial species in homeostatic versus dysbiosis conditions may strongly affect the final host response by altering the balance between anti- and pro-inflammatory LPS.

In the following sections, we analyze the sex-specific factors potentially regulating endotoxin translocation and activity and provide an overview of the human studies investigating the role of metabolic endotoxemia in obesity and related chronic diseases, with special focus on the influence of sex/gender. We propose metabolic endotoxemia as a relevant contributor to gender differences in obesity and in the development of associated chronic diseases. The need to implement sex-based studies and their potential relevance in the identification of sex-specific risk biomarkers useful for prevention are also discussed.

## 2. Sex-Related Regulators of Metabolic Endotoxemia

The increase in circulating LPS levels and the subsequent establishment of a pro-inflammatory milieu leading to metabolic endotoxemia originates from a complex and dynamic interaction among microbial, host, and environmental factors, often exhibiting sex/gender specificity. The gut microbiota (GM) is recognized as the primary source of circulating LPS in metabolic endotoxemia. Alterations in microbial composition or dysbiosis can profoundly influence immune homeostasis and metabolic regulation, thereby contributing to chronic inflammation and driving the onset of various pathological conditions [19]. Sex hormones play a key role in shaping the composition and function of GM and contribute to metabolic and immune homeostasis through the regulation of gut barrier integrity and immune/inflammatory responses [20].

Males normally have higher testosterone levels, and females have higher levels of estrogen and progesterone at reproductive ages; however, hormone levels and regulatory activity can vary based on the reproductive status. Sex-specific differences and hormonal changes give rise to sexually dimorphic microbial profiles, with significant differences in the main bacterial phyla and in the relative abundance of specific genera such as Akkermansia and Prevotella, thus suggesting a different regulation of endotoxemia and inflammatory responses [21,22,23,24]. In this regard, estrogens and progesterone contribute to maintaining gut barrier integrity and restraining chronic inflammation, whereas testosterone is associated with higher basal endotoxin levels and a greater risk of metabolic complications linked to endotoxemia [21,22,23,24]. Therefore, the reduction in sex hormones, as a resultof menopause and andropause, may be associated with metabolic endotoxemia and endotoxin-driven inflammation, and the decline of sex hormone levels in aging is potentially associated with an altered GM. However, it is presently uncertain whether endotoxin levels are associated with an impaired testosterone production in andropause men, while contrasting results were reported in metabolic endotoxemia markers in post-menopausal women.

A conceptual model named “microgenderome” was developed to indicate the interplay among sex hormones, GM, and the immune system, and to elucidate sexual dimorphic susceptibility to diseases [25]. Indeed, sex hormones deeply affect the immune system, which, in turn, is intimately interconnected with GM through a constant and bidirectional signaling process that is essential for maintaining gut barrier function and metabolic/immune homeostasis. The broad distribution of sex hormone receptors across diverse immune cell populations, as well as sex chromosome-associated immune-related genes, leads to consistent differences in immune response between sexes [26]. Generally, testosterone exerts immunosuppressive effects, while estrogens mainly have immunostimulatory properties. As a consequence, women generally mount stronger and more protective humoral and cell-mediated responses at reproductive ages, whereas males exhibit more robust innate immunity activation, including prolonged response to pattern recognition receptor triggering [27].

A bidirectional interaction between GM and visceral AT has also been described. Gut homeostasis and barrier function are influenced by body fat distribution, which differs between men and women, and within women across pre- and post-menopausal stages [28,29]. Men predominantly store visceral AT, generally showing stronger pro-inflammatory responses, whereas women primarily accumulate subcutaneous fat [6]. However, with declining estrogen levels in post-menopausal stages, women experience body fat redistribution towards increased visceral adiposity, which is associated with a reduced Firmicutes/Bacteroidetes ratio and a Proteobacteria-enriched pro-inflammatory microbiota [20,28,29].

Environment and lifestyle factors represent a further layer of regulation and can profoundly impact the microbial, metabolic, and immune balance in a sex and gender-dependent manner. Cani et al. first highlighted that HFD can change the composition of GM by increasing the proportion of Gram-negative Proteobacteria and leading to low-grade inflammation and IR [30]. Alcohol drinking and long-term cigarette smoking also were shown to promote systemic inflammation and oxidative stress by exacerbating gut permeability and LPS translocation, whereas regular physical activity has been associated with enhanced barrier integrity and activation of anti-inflammatory pathways [31,32]. Gender-related psychological and socio-cultural factors, beyond biological sex, can act as facilitators or barriers for healthy behaviors and strongly affect both lifestyle choices and response to lifestyle interventions [33].

**Figure 2:**
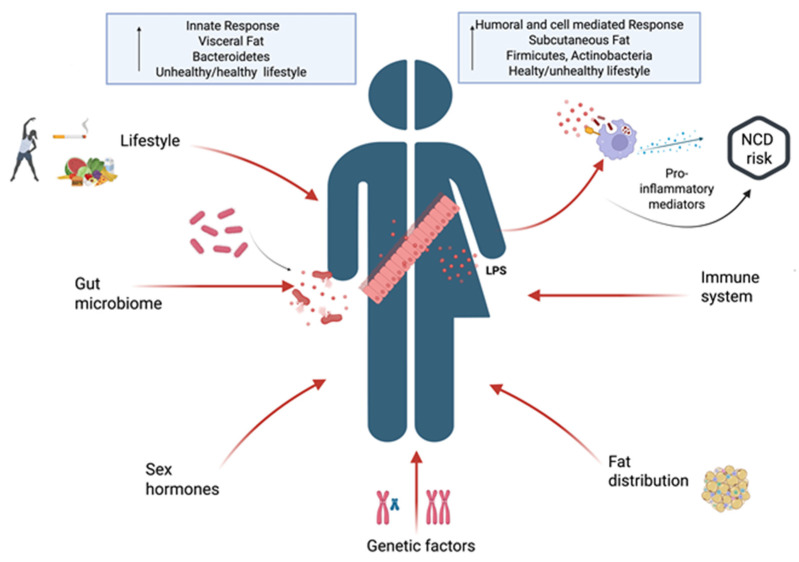
A schematic representation of the sex and gender related factors influencing metabolic endotoxemia. Created in BioRender. Anna Aureli. (2025) https://BioRender.com/ (accessed 5 November 2025).

Overall, the sex/gender specificities in commensal microbial communities, AT distribution and immune responses, together with genetic and lifestyle factors, differently regulate microbial balance and gut barrier integrity, and can affect both the blood translocation and the clearance of endotoxins. This likely results in a sex-dependent regulation of the extent and duration of endotoxin-induced immune activation and, long-term, of the risk of developing chronic diseases. A schematic representation of the sex/gender specific factors influencing metabolic endotoxemia is shown in Figure 2.

## 3. Metabolic Endotoxemia and Inflammation

Subclinical LPS concentrations activate TLR4, leading to the production of numerous pro-inflammatory cytokines and, hence, low-grade systemic inflammation. Thus, metabolic endotoxemia can lead to several chronic inflammatory conditions [19].

As comprehensively documented by Brown, low circulating concentrations of LPS and its related markers LBP and sCD14 can be detected in healthy individuals [34]. Emerging evidence suggests that subtle yet biologically meaningful sex-related variations may partly underlie the transient increases following the ingestion of fat-rich meals and contribute to the different susceptibility of men and women to metabolic and inflammatory disorders [34]. In this regard, the associations between leaky-gut-related markers (zonulin, LBP, sCD14) and metabolic health indicators have been investigated in a large Dutch cohort of 500 adult participants, equally divided by sex, from the Nutrition Questionnaires plus (NQplus) study. Higher zonulin and LBP levels were associated with poorer metabolic profiles and greater inflammation, as indicated by elevated C-reactive protein levels. Notably, sCD14 concentrations were significantly higher in men than in women, whereas zonulin and LBP showed no substantial sex differences [35] (Table 1).

Following fat-rich meals, transient postprandial increases in circulating LPS levels occur, and many studies indicate that diet-induced alterations in GM composition and intestinal barrier function play a key role as mediators of metabolic endotoxemia and its associated disorders. Erridge et al. were among the first to demonstrate that an HF meal can trigger low-grade endotoxemia [36]. This study, involving only healthy men, characterized the kinetics of baseline endotoxin levels in healthy humans, showing that endotoxin is consistently detectable in circulation, with concentrations ranging from 1 to 200 pg/mL. Measurements taken over a 4 h postprandial period further highlighted that acute dietary fat intake can drive detectable increases in circulating endotoxin levels [36]. Subsequent randomized crossover trials have shown that both the amount and type of dietary fat modulate postprandial endotoxin levels. In this regard, the effects of meals rich in ω3, ω6, and saturated fatty acids were investigated in twenty healthy adults of both sexes [37]. The authors showed that postprandial serum endotoxin increased after the meal enriched in saturated fat, decreased after the ω3-rich meal, and remained unchanged after the ω6-rich meal, relative to a control, indicating that fatty acid composition is a critical determinant of endotoxin translocation. Interestingly, despite these variations in postprandial endotoxin levels, systemic inflammatory markers were not significantly altered, suggesting that acute endotoxemia may not always translate into overt inflammation in healthy adults [37]. Additional studies have supported these findings, reporting that mixed meals elicit postprandial increases in endotoxin levels and inflammatory responses in both young and older men and women [38]. They also showed that metabolically healthy older adults do not exhibit an exaggerated postprandial endotoxemia or inflammatory response compared to younger adults, despite differences in oxidative stress–related gene responses [38]. However, in this study, the major outcomes have not been stratified by sex. More recently, Bowser et al. demonstrated, in 13 healthy young men, that consuming an isocaloric HFD for five days increased fasting endotoxin levels independently of changes in gut permeability. This rise in endotoxemia was accompanied by alterations in skeletal muscle metabolism, including enhanced postprandial fat oxidation, without affecting body weight or insulin sensitivity [39]. Furthermore, in a study specifically in women, Al-Disi et al. observed that postprandial endotoxemia occurs regardless of IR status, underscoring that this response is not limited to metabolic disease and may be modulated by sex-specific factors [40]. Despite this accumulating evidence, the data on gender differences in postprandial endotoxemia remain limited, and in none of the major trials have they been stratified by gender. This represents a critical gap, as sex-related differences in postprandial metabolism may influence not only the extent and kinetics of meal-induced endotoxin translocation but also its potential contribution to chronic low-grade systemic inflammation and cardiometabolic risk.

Systemic inflammation can be experimentally elicited in healthy humans by intravenous administration of low doses (0.2–2 ng/kg body weight) of purified bacterial endotoxin. The human endotoxemia model can be exploited, in a highly standardized and reproducible manner, for the study of complex disease inflammatory responses and their modulation in vivo [41]. Controlled endotoxin challenge studies have revealed clear sex differences in immune responses to LPS exposure, although the results obtained have been inconsistent across studies and dependent on the context (Table 1). Following in vivo LPS injection, women mount stronger and faster pro-inflammatory responses than men, evidenced by higher circulating concentrations of TNF-α, IL-6, and IL-1β, as well as enhanced febrile and neuroendocrine reactions [42,43,44,45]. These sex differences develop within hours and arise from a rapid hormone-driven innate immune signaling. Estrogens amplify responses by enhancing TLR4 signaling, Nuclear factor kappa-light-chain-enhancer of activated B cells (NF-κB) activation, and endothelial responsiveness, whereas androgens tend to reduce cytokine production [43,45]. However, in a recent study, the endotoxin challenge in female versus male sex was not associated with greater increases in activation of transcription factors involved in the inflammatory response, except for a higher endotoxin-induced activity of cAMP response element-binding protein (CREB) [46]. Furthermore, Jansen et al. have confirmed that women exhibit markedly higher plasma concentrations of pro-inflammatory cytokines than men in response to LPS challenge, but the observed differences in cytokine production did not arise from distinct underlying molecular pathways, and were strongly influenced by sex hormones, supporting an important immunoregulatory role for these mediators [47]. Other in vivo studies, however, did not reveal sex differences in immune parameters [48] or showed greater LPS-induced cytokine production in men as compared to women [49]. However, these sex differences disappeared after normalization for monocyte number, except for a lower IL-10 level in men [49].

Similarly, ex vivo assays, using whole blood or purified monocytes, showed higher LPS-stimulated TNF-α and monocyte-derived cytokine production in men compared to women [50]. Otherwise, in a cohort of 160 adults (67% women), although no significant sex differences were detected in the levels of endotoxemia markers, distinct sex-specific patterns emerged in how these markers influenced the association between depressive symptoms and inflammation [51]. Specifically, in men, higher depressive symptoms were related to heightened ex vivo inflammatory responses to LPS, whereas in women, higher depressive symptoms were related to attenuated inflammatory responses. At lower endotoxemia levels, no significant associations were observed in either sex [51]. Although this discrepancy is likely due to the higher LPS concentrations used in ex vivo studies, it could also reflect the influence of factors that amplify women’s inflammatory responses in vivo but are absent in ex vivo assays, emphasizing that sex differences in innate immunity are context-dependent and may depend on multiple interacting biological and environmental factors. In this regard, Ferguson et al. investigated whether ethnicity, in addition to gender, influences the inflammatory response to an acute LPS challenge. They reported the novel finding of differences in LPS-induced inflammatory responses in individuals of African versus European ancestry. Gender differences; however, were modest and less consistent; males exhibited a slightly higher C-reactive protein response than females, indicating that, in this context, ethnicity may be a stronger determinant of evoked inflammation than gender [52].

Overall, although basal endotoxemia remains low in healthy individuals, the reported evidence points to sex-related differences in gut permeability, post-prandial lipid handling, and response to endotoxins, all factors known to modulate the inflammatory response.

## 4. Metabolic Endotoxemia in Obesity and the Impact of Diet

The increased prevalence of obesity is a major health problem afflicting, nowadays, adults and children worldwide (WHO, Obesity and Overweight. Fact sheet 2025. Available online at: http://www.who.int/mediacentre/factsheets/fs311/en/ (accessed on 20 November 2025)). Obesity is a chronic inflammatory state driven by the interaction of genetic susceptibility and environmental factors [53]. The related persistent low-grade inflammation can arise through a range of mechanisms and, according to the prevailing hypothesis, endotoxemia not only contributes to obesity development but is considered a key mediator of associated metabolic derangements [4,53]. More recently, being overweight, a condition of excessive fat deposits, was also associated with the risk of several serious health problems, including T2DM, heart disease, stroke, high blood pressure, and certain types of cancer [54]. Evidence for metabolic endotoxemia in overweight individuals was widely reported [4].

Similarly to mouse models [11] current hypotheses indicate that, also in human obesity, the GM serves as the link between HF feeding, endotoxin translocation, and systemic inflammation. In this regard, Ley et al. were the first to describe differences in GM composition of obese compared to lean individuals [55]. At the phylum level, individuals with obesity had a higher Firmicutes/Bacteroidetes ratio than age-matched controls, and this difference was reversed with either carbohydrate-restricted or fat-restricted diets, although opposite results were also reported [55,56]. Overall, evidence showed that obesity is associated with phylum-level changes in the microbiota, reduced bacterial diversity, and altered representation of bacterial genes and metabolic pathways [57]. The range of endotoxin concentrations reported in healthy individuals, obesity, and obesity-associated metabolic disorders varies widely in the literature [58,59,60,61,62,63]. However, cross-sectional analyses have consistently shown that patients with obesity have higher circulating LPS levels compared to their lean counterparts, and that LPS levels in both sexes are positively correlated with Body Mass Index (BMI), waist circumference, and other measures of adiposity, suggesting a link between endotoxemia and the degree of obesity [4]. Specifically, in a –case–control study from China, LBP levels were significantly higher in overweight and obese patients than in normal-weight individuals [64]. Similarly, in a study including Spanish volunteers with morbid obesity or with glucose intolerance, as well as in a Finnish nutritional study, high serum LPS activity was significantly associated with prevalent obesity and positively correlated with increasing BMI [65,66,67]. This finding aligns with the link among obesity, high LBP concentrations, and metabolic derangement reported in different studies involving adults with metabolic syndrome (MetS) [68], an adolescent population with IR [69] or T2DM patients [70]. Interestingly, higher plasma LPS concentrations were associated with less favorable phenotype in overweight/obese men [71].

Overall, these studies have shown that the elevation in circulating endotoxin observed in individuals with obesity is reproducible after adjusting for ethnicity, sex or age of the study group and is related to metabolic abnormalities. However, they did not report stratified findings for men and women, thus leading to limited available evidence on potential sex differences. Likewise, other studies have focused on one sex only, reporting data on specific subgroups of women. Metz et al. recently showed that, in a cohort of women matched for age and having self-declared hypercholesterolemia and/or hypertension, overweight individuals exhibited a pro-inflammatory state, as indicated by increased plasma concentrations of LBP, IL-6 and other pro-inflammatory mediators and decreased anti-inflammatory adiponectin levels, compared with lean controls [72]. Similarly, a study in Gambian women showed evidence for metabolic endotoxemia in participants with obesity and T2DM, with LPS levels being highest in the obesity/diabetic group compared with the lean and obesity/non-diabetic groups [73]. Plasma LBP concentration was also positively associated with increased visceral AT in post-menopausal women, although no differences in markers of endotoxemia between pre- and post-menopausal women were reported in previous studies [74]. Regarding pregnant women, twofold higher endotoxemia levels were observed in obese compared to lean individuals, with pre-gravid obesity associated with increased maternal endotoxemia and metabolic inflammation [60]. These findings highlight a significant correlation among endotoxemia, obesity, and inflammatory measures in women.

Although limited, some studies have analyzed the link between obesity and endotoxemia, focusing on sex differences (Table 1). Gonzalez-Quintela et al. [68] have investigated LBP concentrations and their associations with demographics, lifestyle factors, and common metabolic abnormalities in adult males and females. They found that individuals with overweight or obesity, as well as individuals having MetS, had higher serum LBP concentrations than lean healthy individuals, with a significant trend towards increased LBP concentrations with increasing categories of BMI and with age. In particular, LBP concentrations were independently associated with abdominal obesity and low HDL-cholesterol. Importantly, the male sex tended to be independently associated with higher serum LBP concentrations [68]. In line with this result, in a population-based cross-sectional study of men and women from three different ethnic groups (n = 193, aged 40–59 years), endotoxin levels were higher in men than in women, in association with obesity measures. For waist circumference, the association was significant in men but not in women. Furthermore, the sex differences observed in endotoxin levels are consistent with the sex specificities in vascular risk [75]. On the other hand, a study conducted on young individuals (n = 32, aged 18–25 years) with obesity revealed a trend toward higher LPS levels in women than in men. Moreover, LPS concentrations showed a positive correlation with BMI, waist circumference, and triglycerides in women, possibly reflecting enhanced fat absorption and storage capacity as compared to men [76]. Accordingly, when subjects with obesity were classified based on LPS levels, women were prevalent (75%) in the high LPS group, while the low LPS group was equally distributed between sexes [77]. These discrepancies could be, at least partly, explained by the different ages of women participating in the different studies.

Unhealthy diets can modify the composition of GM, increase intestinal permeability, and reduce LPS clearance, thus favoring metabolic endotoxemia and inflammatory response [78]. As already reported, studies investigating the importance of nutrients for endotoxin absorption in humans have focused mainly on healthy individuals [16]. However, whether diet can affect endotoxemia in the different metabolic disease phenotypes, including overweight, is an understudied phenomenon [79]. Overall, post-prandial endotoxemia is differentially modulated by the amounts of ingested fat in individuals with obesity compared to their lean counterparts, showing a positive association between fat caloric doses and increased LPS levels [79,80]. Obesity appears to amplify the impact of HF diets on metabolic endotoxemia by impairing the clearance of chylomicron-bound LPS, which contributes to sustained systemic inflammation and the development of IR [79]. In this regard, it has been demonstrated that smaller chylomicrons stay longer in the bloodstream in people with obesity, lowering the effectiveness of LPS clearance. Lean individuals, on the other hand, have larger chylomicrons that are more efficiently processed by the liver, thereby minimizing endotoxin exposure [81].

These findings further suggest that the relationship between dietary fat intake and metabolic endotoxemia is influenced by body weight status. Accordingly, among morbidly obese adults, the ingestion of a fat overload increased the levels of LPS in participants with the highest postprandial hypertriglyceridemia [77]. Metabolic endotoxemia was also exacerbated after dietary fat intake among overweight individuals. Al-Disi et al. [40] demonstrated that an HF meal exacerbated postprandial endotoxemia in normal-weight and overweight participants in a cohort of Saudi women.

To date, only a few studies have investigated the long-term effect of diet on postprandial and fasting levels of LPS in individuals with obesity. A transient increase in circulating LPS levels can be induced by a wide variety of high-energy/HF diets, particularly those lacking in healthful dietary components [16]. On the contrary, diet-induced weight loss, adopting a healthy diet, or reducing caloric intake have been shown to reduce circulating endotoxin and metabolic perturbations in individuals with obesity [4,82]. Consistently, bariatric surgery, an effective obesity treatment option that results in stable weight loss and improvement of obesity-related conditions, is associated with a reduction in circulating LPS levels, along with altered GM, reductions in pro-inflammatory pathways, and improvements in glucose homeostasis [83]. Changes in LPS, LBP, and LPS signaling components after varying types of bariatric surgery are reported, further supporting the link between obesity and endotoxemia [83]. However, the exact relationship between dietary habits in conditions of obesity and circulating endotoxin levels is still inconsistent [16].

Another limitation of these studies is that most dietary interventions have been conducted on male groups, while the effects of bariatric surgery have been investigated predominantly in women, in line with the higher adherence of women to the surgical intervention. Very few studies have looked at both sexes. Because of the heterogeneity of available studies and the little consideration for sex as a biological variable, it remains unclear to what extent the regulatory effects of diet on endotoxemia could be associated with sex, although indirect evidence exists. In this regard, sex hormones have been described to directly affect GM in obesity [84]. Males with obesity have lower species richness, and testosterone has been found to be associated with increased anti-inflammatory butyrate-releasing Firmicutes. Accordingly, serum LBP was negatively correlated with testosterone but positively correlated with IL-6 in men with obesity [85]. On the other hand, females with obesity, despite having greater microbial diversity, have increased estradiol and Bacteroidetes abundance, resulting in greater LPS release and greater immune response elicited [84]. On this point, post-menopausal reductions in estrogen can diminish this protective effect, narrowing sex disparities with advancing age. Nevertheless, it has been recently reported that serum endotoxin concentrations increase following a HF diet or HF meal intake in individuals with obesity, with females (mean age of 64 years) showing higher levels compared to the male group [86]. Future work should prioritize longitudinal, sex-stratified studies to fill this gap in knowledge. Specifically, more studies are necessary to deepen the understanding of how specific nutrients or dietary interventions may affect metabolic endotoxemia and how the effects of diet on endotoxemia and inflammation are influenced by sex hormones and GM composition.

**Table 1:** Sex differences in metabolic endotoxemia in inflammation and obesity.

Study	Population/ Sample Size	Males/Females	Age (Years)	LPS Stimulation/ Endotoxemia Assessment	Results	References
***Metabolic endotoxemia and inflammation***						
**Observational**	N = 500 Caucasian ethnicity	250 M, 250 F	20–70	LBP determination	LBP, Zonulin: F = M sCD14: ↑ F	Hoshiko et al. (2021) [35]
**In vivo LPS challenge**	N = 28 Caucasian ethnicity	14 M, 14 F	22–41	LPS = 0.4 ng/kg	LBP: ↑ F TNF-α, IL-6, IL-1RA: ↑ F sCD14: F = M IL-10: ↓ F	Engler et al. (2016) [42]
	N = 30 Ethnicity not specified	15 M, 15 F (9/15 F on hormonal contraceptives)	25–27	LPS = 2 ng/kg	- Baseline levels LBP: ↑ F (9.9 ± 1.1 vs. 7.0 ± 0.8 pg/mL, *p* < 0.042), - After LPS LBP: ↑ F (32.9 ± 4.2 vs. 21.1 ± 1.9; *p* < 0.004) TNF-α, IFN-γ, CRP: ↑ F IL-6, IL-10: F = M	van Eijk et al. (2007) [43]
	N = 40 Ethnicity not specified	20 M, 20 F on hormonal contraceptives	18–45	LPS = 0.4 ng/kg	TNF-α, IL-6: ↑ F	Wegner et al. (2015) [44]
	N = 20 Ethnicity not specified	10 M, 10 F on hormonal contraceptives	22–41	In vivo-LPS = 0.4 ng/kg Ex vivo-LPS = 12.5–400 pg/mL	In vivo: TNF-α, IL-6: ↑ F Ex vivo: TNF-α, IL-6: F = M	Wegner et al. (2017) [45]
	N = 59 Different ethnicity	22 M, 37 F	18–50	LPS = 0.8 ng/kg	TNF-α, CXCL8, FOS, JUN, CCL3, ADRB2, CREB: ↑ F NF-κB, AP-1: F = M	Boyle et al. (2024) [46]
	N = 100 Ethnicity not specified	56 M, 54 F (24/54 F on hormonal contraceptives)	18–34	LPS = 1 ng/kg At day 0 and day 7	TNF-α, IL-6, IP-10; G-CSF: ↑ F	Jansen et al. (2025) [47]
	N = 24 Different ethnicity	16 M, 8 F	26–29	LPS = 2 ng/kg	TNF-α, IL-6, CXCL8, IL-10: F = M	Coyle et al. (2006) [48]
	N = 294 Different ethnicity	143 M, 151 F	18.5–36	LPS = 1 ng/kg	- Baseline IL-6, IL-1RA: ↑ F; ↑ Africans vs. Europeans - After LPS IL-6, IL1-RA: ↑ F IL1-RA: ↓ Africans vs. Europeans	Ferguson et al. (2013) [52]
**Ex vivo LPS stimulation**	N = 4020 Different ethnicities from 15 study populations	F, M (M~42%)	21–85	LPS = 10 ng/mL–10 µg/mL	TNF-α, IL-6, IL-12, IL-1β IL-1RA, IL-10: ↓ F IFN-γ: F = M GM-CSF: ↑ F After adjusting for monocyte count IL-10: ↓ F	Beenakker et al. (2020) [49]
	N = 231 Different ethnicity	159 M, 72 F	Mean age F: 30.9 ± 8.2 M: 30.4 ± 8.7	LPS = 1 μg/mL	TNF-α: ↓ F	Moxley et al. (2002) [50]
	N = 160 Different ethnicity	53 M, 109 F (36/109 F post-menopausal)	25–65	LPS = 1 μg/mL	At lower LBP:sCD14 levels depressive symptoms and inflammation: F = M At higher LBP: sCD14 levels depressive symptoms and inflammation: ↓ F	Knight et al. (2020) [51]
***Metabolic endotoxemia and obesity***						
**Cross-sectional**	N = 420 (Nw: 112; Ow: 180; Ob: 127) Caucasian ethnicity	189 M, 231 F	18–92 (mean age 55)	LBP and sCD14 determination	sCD14: ↓ F (F = 3.28/M = 3.30 µg/mL) LBP: - ↓ F (F = 6.97/M = 7.39 µg/mL) - ↑ Age (>50) - ↑ obesity, overweight, MetS - Positive correlation with serum IL-6, CXCL8 and sCD14 levels	Gonzalez-Quintela et al. (2013) [68]
	N = 193 Different ethnicities: - Caucasians (61); - Africans (68); - South Asians (63).	96 F, 97 M - Caucasians (30 F/31 M) - Africans (33 F/35 M) - South Asians (33 F/30 M)	40–59	LPS serum levels	LPS: - ↓ F (Caucasian: F = 9.6/M = 12.5 EU/mL; African: F = 9.1/M = 11.1 EU/mL; South Asian: F = 12.3/M = 14.2 EU/mL) - ↑ obesity, overweight, MetS - ↑ Africans vs. Caucasians vs. South Asians origin, - In men: positive correlation LPS/waist circumference	Miller et al. (2009) [75]
	N = 64 (Nw: 32; Ob: 32) Ethnicity not specified	Nw: 16 M, 16 F Ob: 13 M, 19 F	18–25 (mean age 21)	LPS serum levels	LPS: - ↑ F (F = 1.20/M = 1.16 EU/mL) - In women: positive correlation LPS/waist circumference	Radilla-Vázquez et al. (2016) [76]
	N = 33 (Ob) (Low LPS: 17; High LPS: 16) Ethnicity not specified	Low LPS: 47.1% M: 52.9% F High LPS: 25% M/75% F	Mean age Low LPS: 45.27 ± 2.39 High LPS 43.40 ± 2.71	LPS serum levels	Low LPS group (0.081 ± 0.003 EU/mL): - F/M = 52.9/47.1% High LPS group (0.315 ± 0.033 EU/mL): - F/M = 75/25%	Clemente-Postigo et al. (2019) [77]
**Intervention (5 days of HFD)**	N = 32 16 Nw 16 Ob	66% F	50–79	LPS serum levels	LPS: - Nw: ↑ F (F = 0.28/M = 0.14 EU/mL) - Ob: ↑ F (F = 0.40/M = 0.32 EU/mL)	Ogilvie et al. (2025) [86]

EU, endotoxin units; F, females; HFD, high-fat diet; LPS, lipopolysaccharide; LBP, LPS binding protein; M, males; MetS, metabolic syndrome; Nw, normal weight; Ob, obese; Ow, overweight; sCD14, soluble CD14; vs., versus; ↑↓ F, higher/lower in females than males.

## 5. Metabolic Endotoxemia in Obesity-Associated Noncommunicable Diseases

Studies in mice and humans suggest that chronic/sustained serum levels of microbiota-derived LPS, as observed in obesity, represent a triggering factor for metabolic disorders and increase the risk of several NCDs [5,87]. A summary of the human studies investigating the link between metabolic endotoxemia and the risk of developing chronic diseases from a sex perspective is reported in Table 2.

### 5.1. Metabolic Disorders

T2DM is a group of metabolic disorders characterized by hyperglycemia whose incidence has increased during recent decades, especially in Western countries, alongside short- and long-term complications, thus accounting for high social and economic burdens [88]. Overweight and obesity, gut dysbiosis, and chronic inflammation represent the main risk factors. Evidence, largely from animal studies, indicates that endotoxin translocation into the bloodstream contributes to the sub-clinical inflammation observed in T2DM [30]. Results from a systematic review analyzing ten human studies revealed that, despite a great variability in the estimates of metabolic endotoxemia, higher LPS or LBP concentrations are detected in diabetic subjects compared to healthy controls in all studies [89]. Endotoxin levels were also increased in subjects with impaired glucose tolerance compared to healthy controls [40]. Furthermore, blood LPS levels were positively associated with the risk of developing T2DM in a Finnish population-based prospective cohort [90].

Men exhibit a higher incidence of T2DM than women; however, no specific gender-related trends in T2DM-associated endotoxemia have been described so far, as most studies included only male or female subject groups and no disaggregated results were reported when both sexes were analyzed. In a more recent study involving T2DM and control subjects from an Arabian population, significantly higher circulating levels of LPS were detected in patients compared to controls, and this increase turned out to be sex independent [91]. In addition, diabetic subjects presented higher fasting and postprandial LPS concentrations compared to lean non-diabetic and obese individuals [40,62]. Accordingly, the cardiometabolic risk profile is higher in T2DM subjects than in either of the overweight or the non-diabetic lean control subjects [40]. In general, the circulating levels of LPS after a single HF meal appear to be more pronounced in metabolically impaired individuals [16]. Moreover, a high postprandial endotoxemia was associated with an increased risk of T2DM among overweight/obese individuals in the CARDIOPREV longitudinal study, highlighting the risk predictive value of blood LPS [92]. The impact of antidiabetic medication on endotoxemia was also reported, with lower blood LPS levels detected in subjects in therapy with oral hypoglycemics and/or insulin [89].

Obesity and insulin resistance are intimately related to the metabolic dysfunctions observed in MetS. Cross-sectional associations of serum LBP levels with MetS have been reported [68,93,94]. Among MetS components, LBP was independently associated with abdominal obesity and low HDL-cholesterol, whereas no sex-related differences were observed [68]. In addition, two large-scale prospective studies involving Asian populations addressed the positive association of serum LBP with the risk of developing MetS [95,96]. Baseline LBP levels were found to strongly associate with all MetS components and with inflammatory markers, although no sex-related results were reported.

In the context of NCD, metabolic dysfunction-associated steatotic liver disease (MASLD) is emerging as a critical contributor to the global burden, being strictly connected to obesity, IR, and T2DM. In line with the crucial role of chronic inflammation in metabolic disorders, serum endotoxin levels were found to be significantly higher in patients with biopsy-proven MASLD compared to controls, and increased sCD14 levels were associated with the severity of fibrosis [97]. Moreover, LPS levels were significantly reduced in patients treated with anti-obesity drugs in association with reduced body weight and liver enzymes, strengthening the evidence that the control of body weight and adiposity results in lower inflammation and rescue of metabolic homeostasis [97]. Future prospective population-based studies and randomized controlled trials, including sex balanced cohorts, will help fully address the gender specificities in the link between endotoxemia and the risk of metabolic disorders, as well as in the efficacy of LPS-lowering medication or dietary interventions in the prevention of endotoxemia.

### 5.2. Cardiovascular Diseases

CVD includes a wide range of disorders of the heart and blood vessels, such as coronary heart disease (CHD), cerebrovascular disease, aortic atherosclerosis, and peripheral artery disease, most of which exhibit higher incidence or earlier onset in men compared to women. Conversely, in post-menopause, women display higher CVD rates and an increased risk of cardiovascular mortality [98]. Modifiable CVD risk factors, such as unhealthy diets, physical inactivity, overweight/obesity, and T2DM, are all associated with alterations in GM, making the gut-heart axis a main actor in CVD and a potential therapeutic target. Accumulating experimental and epidemiological evidence indicates that chronic inflammation associated with low-level subclinical endotoxemia is a determinant in CVD [99]. Endotoxins are pro-atherogenic molecules and may trigger or accelerate atherosclerosis through multiple mechanisms, including increased Reactive Oxygen Species (ROS), chemotactic and pro-inflammatory cytokines and adhesion molecules. Notably, there is evidence that the lipid-lowering statins, largely used in CVD prevention, can also attenuate the effects of endotoxin on immune and endothelial cells [100].

The association of endotoxemia with the risk of atherosclerotic disease was initially described in a random population of 516 men and women [101]. These findings were supported and extended in larger prospective studies where baseline serum LBP levels were significantly associated with incident cardiovascular events [102,103]. Moreover, serum endotoxin was proposed as a reliable early marker for obesity-related cardiovascular injury in young people [104]. Additionally, an observational prospective study on a cohort of atrial fibrillation patients highlighted a significant association between blood LPS and the risk of major adverse cardiovascular events, with patients in the highest tertile of LPS (>100 pg/mL) showing the highest risk [105]. In this study, blood LPS concentrations were also inversely associated with adherence to the Mediterranean diet, further supporting the link between endotoxemia and lifestyle and the need to explore nutritional/lifestyle interventions in atrial fibrillation, and more generally, in the primary prevention of CVD.

Sex disparities have been described in CVD incidence and prognosis, and sex-dependent endotoxemia levels and immune response to endotoxins have been hypothesized as contributing factors. Although the link between endotoxemia and CVD has started to be dissected from a gender perspective, no definitive results are available yet. In a cross-sectional study of 196 patients with T2DM, serum LBP correlated with arterial stiffness, pointing to blood LBP as a biomarker of subclinical atherosclerosis. Despite comparable blood LBP levels being detected in women and men, a significant association with arterial stiffness was selectively observed in men, consistent with their higher atherosclerosis risk [106]. Conversely, in the UK Wandsworth Heart and Stroke cross-sectional study, involving 192 participants of different ethnicities, blood LPS levels were significantly lower in women than in men, according to the sex differences in vascular risk, and were positively associated with waist circumference and waist-to-hip ratio [75]. Furthermore, a graded increase in endotoxin levels from black African to white to South Asian participants was observed, consistent with the graded increase in estimated CHD risk [75]. Although not evidenced by the authors, the different mean age of the participants, likely reflecting different pre-/post-menopausal women ratios, as well as the presence of comorbidities, might explain the discrepancy in endotoxemia levels observed in the latter studies. In this regard, in a recently reported clinical trial, sex-associated plasma LPS levels were measured in T2DM and non-T2DM overweight/obese participants aged 20–75 years who had experienced previous coronary events. When analyzing the non-diabetic group, LPS concentrations, correlating with the number of atherosclerotic plaques, were found to be lower in women than in men. In contrast, the sex differences flattened out in the group of subjects with T2DM [107]. The link between plasma LBP or sCD14 and incident CHD was recently investigated in two prospective nested case-control studies, the Health Professionals Follow-up Study, including 496 men, and the Nurses’ Health Study II involving 212 women pairs. While LBP levels were found not to be associated with CHD in either sex, higher levels of sCD14 correlated with an increased CHD risk in female participants [108].

Childhood obesity has a significant impact on future chronic inflammation and CVD risk. In this regard, Varma et al. reported that, in a cohort of 60 obese children and adolescents, serum LPS levels were significantly higher in females compared to males and strongly correlated with markers of atherogenesis and vascular injury, pointing to endotoxins as potential biomarkers of sub-clinical inflammation and early CVD risk [109].

### 5.3. Gastrointestinal Cancers

Overweight/obesity and unhealthy lifestyles also represent risk factors for different cancers, mainly gastrointestinal cancers, and a role for bacterial endotoxins in their promotion has been highlighted [87,110]. Altered microbiota composition and impaired gut barrier function have been repeatedly described in colorectal cancer (CRC), the most common gastrointestinal tumor, which exhibits gender specificities in both incidence and prognosis [111]. LPS was shown to both activate cancer-promoting pathways and favor tumor immune evasion in animal models, and its trapping in experimental CRC models was associated with relieved immunosuppression and boosted immunotherapy response [112].

The impact of endotoxemia on human cancer has been analyzed by measuring circulating LPS or LPS-associated markers in patients with CRC or pre-cancerous lesions. In a cross-sectional study including colon adenoma cases and non-adenoma controls, significantly higher plasma concentrations of endotoxin, as well as of TNF-α, were found in cases compared to controls, and circulating LPS was positively associated with polyp severity [113]. Moreover, in a cohort of subjects with colorectal adenoma, blood anti-LPS antibody concentrations were found to be higher among men as compared to women, and to positively correlate with BMI [114].

In other studies, the risk predictive potential of markers of endotoxemia was investigated in both sexes, given the higher incidence of CRC in men compared to women. In a nested study including 1065 incident CRC cases and matched controls from the European Prospective Investigation into Cancer and Nutrition Cohort, no association between baseline anti-LPS antibodies and CRC risk was found when the whole population was analyzed. However, analyses stratified by sex showed a statistically significant positive association for men [115]. Conversely, no sex-specific association was observed between baseline plasma LBP concentrations and incident cases of colon cancer in a multiethnic study, alongside comparable LBP concentrations in women and men [116]. The hormonal status of female participants might have contributed to the different outcomes reported in the two studies. Over 20% of women enrolled in the former study were pre-menopausal or on hormone replacement therapy, whereas no information, except the higher mean age, was provided in the latter.

### 5.4. Neurodegenerative Diseases

Individuals with obesity often show a higher prevalence of mental health disorders compared to normal-weight subjects. The alteration of the GM observed in obesity significantly influences mental health via the gut–brain axis, a complex, bidirectional communication network linking the central nervous system and the gastrointestinal tract, which highlights how the gut and brain are deeply interconnected and influence each other in ways that affect overall health and mental well-being [117].

Research has increasingly focused on the connection between obesity and ND, particularly Alzheimer’s (AD) and Parkinson’s (PD) diseases, multiple sclerosis, and amyotrophic lateral sclerosis, representing a growing cause of disability in developed countries. Obesity-induced metabolic endotoxemia has been proposed as a key factor linking GM alterations to neuroinflammation. Several studies using animal models revealed that elevated LPS levels in the context of obesity can both significantly compromise the blood–brain barrier’s integrity by damaging tight and adherent junctions and activate microglia to release inflammatory and pro-oxidant mediators, that finally lead to neuronal damage and loss. Detailed mechanisms linking obesity and microbiota-derived endotoxins with neuroinflammation have been recently reviewed [118].

An increased proportion of LPS-producing Enterobacteriaceae and a higher Gram-negative to Gram-positive bacteria ratio, correlating with motor symptoms, were observed in the gut of PD patients compared to age-matched controls. As the gut–brain axis is emerging as a relevant factor, blood endotoxin levels have been estimated in PD patients [119,120]. Higher LPS concentrations were found in patients compared to age-matched controls, particularly in subgroups suffering from constipation, sometimes in association with immune activation and increased risk of dementia or with higher intestinal alpha-synuclein staining [121,122,123,124]. Conversely, comparable or lower endotoxin concentrations were reported in other studies [125]. Increased levels of LBP in the plasma and cerebrospinal fluid of PD patients were also shown in case-control studies, and an endotoxin hypothesis was formulated for PD [126,127]. In line with this hypothesis, a link between LBP levels in pre-diagnostic plasma samples and the risk of developing PD has been recently determined in a nested study from a large prospective European cohort [128]. Notably, the association was found statistically significant only among overweight/obese individuals, and in women, but not in men [128]. While previous studies mostly included subject groups with male prevalence or did not report sex and body weight disaggregated results, Zhao et al. highlighted the contribution of body weight and sex in endotoxin-induced inflammation, thus strengthening the need for selective prevention strategies [128].

Although PD incidence is higher in men, women and obese individuals experience significantly higher prevalence and severity of chronic pain, a common non-motor symptom [129]. In this regard, both peripheral and central inflammation have emerged as potential mechanisms driving pain development, and a chronic dysregulated inhibitory response to pain is common for several idiopathic pain syndromes, linked to inflammation and showing female predominance [130,131,132]. In a randomized controlled human study using low doses of LPS (0.6 ng/kg) injection as an experimental model of systemic inflammation, deep pain was found to strongly correlate with LPS-induced immune activation, and this effect was restricted to women [133]. Conversely, a different randomized controlled crossover study involving 40 healthy subjects (male/female 1:1) receiving a lower (0.4 ng/kg) LPS dose did not highlight any difference between sexes in pain sensitivity, despite the stronger inflammatory response and higher cortisol rise observed in females [44]. In both studies, however, the response to an acute LPS challenge was analyzed as experimental endotoxemia in human volunteers is limited by ethical considerations to low LPS dose and short exposure.

Endotoxin-induced neuroinflammation has also been associated with AD, a progressive neurodegenerative disorder primarily affecting memory and thinking, and one of the main causes of dementia, morbidity, and death worldwide. Post-mortem analyses of AD patients revealed persistently elevated LPS levels in the brain, particularly in the areas vital for memory and cognition, like the hippocampus, suggesting that chronic exposure to circulating endotoxins may contribute to inducing or worsening the neurodegenerative processes linked to AD [118,134,135]. Consistent with this hypothesis, acute intravenous LPS injection in healthy male volunteers induced symptoms overlapping with AD, such as anxiety and depressed mood, and impaired declarative memory, alongside systemic inflammation and microglia activation [136,137,138]. LPS concentrations in blood and cerebrospinal fluid have been measured in AD patients in comparison with healthy controls. Despite differences in the absolute endotoxin concentrations, 3- to 6-fold higher levels were found in AD patients in all studies, correlating positively with markers of gut permeability, systemic immune activation, and brain pathology, and negatively with the mental state of the participants [123,139,140,141,142]. Notably, increased endotoxin levels were also reported in subjects with mild cognitive impairment, further supporting the involvement of metabolic endotoxemia in AD development [140].

Furthermore, in a recent pilot study, the reduction in circulating LPS by means of a probiotic has been hypothesized as a perspective for AD treatment. A decrease in serum LPS was obtained in 40 patients with mild AD after a 6-month treatment with the probiotic, alongside improved Mini-Mental State Examination scores [143]. Despite the sex-balanced study group, no sex-specific results were reported. Women account for two-thirds of AD dementia diagnoses. Due to their longer life expectancy, the lifetime risk is greater for women, but gender related factors also affect AD prevalence. Nevertheless, most studies included subject groups of all men or with a predominance of the male sex, and gender-related differences were not analyzed, even when sex-balanced enrollment was performed. Future prospective longitudinal studies, including sex disaggregation of results and paying attention to the role of body weight and lifestyle, will help better clarify the contribution of endotoxin-induced chronic immune activation and the gender specificities in the development of ND, thus opening the way for more personalized prevention and treatment [16].

The link between microbiota-derived LPS and other inflammation-based neurodegenerative disorders has been described in animal models [118]. In humans, higher levels of LPS were described in plasma from patients with sporadic Amyotrophic Lateral Sclerosis as compared to healthy controls, showing a positive correlation with the degree of blood monocyte activation and a negative association with IL-10 concentrations [123,139].

### 5.5. Aging

Aging is a risk factor for most metabolic disorders and ND. Low-grade inflammation, resulting from altered microbiota composition, impaired gut barrier function, and microbial translocation, was reported to increase with age (inflammaging) and to strongly affect the health status [144]. In this regard, circulating endotoxin levels have been shown to act as predictors of telomer length shortening, suggesting a role for endotoxemia, likely arising from chronic consumption of unhealthy diets, in accelerating biological aging and disease onset [91]. In a recent study, age relationships with markers of gut barrier function, systemic inflammation, and immune cell activation were investigated in a sex-balanced healthy population of 95 participants aged 20–100 years [145]. Sex differences in gut barrier function were reported, with females showing an increased epithelial permeability throughout life as compared to males. Furthermore, females displayed an age-associated increase in circulating bacterial products, including LPS, alongside increased markers of inflammaging, like blood monocytes and TNF-α [145]. These findings confirm the sex specificities in circulating levels and response to endotoxins and reinforce the need for gender-specific prevention strategies for chronic diseases.

**Table 2:** Sex differences in metabolic endotoxemia in obesity-related diseases.

Disease Condition	Study Type	Population/ Sample Size	F/M (%F)	Age (Range/ Mean)	Endotoxemia Assessment	Results		References
						ME Markers (Blood Levels)	ME Markers (Association with Disease or Disease Risk)	
***Metabolic disorders***								
**T2DM**	Cross-sectional	Saudi Arabian cohort/ T2DM (N = 387) Controls (N = 388)	T2DM: 186 F/201 M Controls: 214 F/174 M (51.7)	40–80	Serum LPS	↑ in T2DM M = F	ND	Al-Daghri et al. (2022) [91]
**MetS**	Cross-sectional	Spanish cohort/ random sample of the adult population (N = 420) MetS (N = 105) Controls (N = 315)	231 F/189 M (55)	18–92	Serum LBP	↑ in MetS M = F ↑ in Ow, Ob, and older subjects	positive association with MetS M = F positive association with central obesity, low HLD-cholesterol, liver enzymes, IL-6, IL-8	Gonzalez-Quintela et al. (2013) [68]
***Cardiovascular diseases***								
**Subclinical atherosclerosis** **(arterial stiffness)**	Cross-sectional	Japanese cohort/ T2DM (N = 196)	95 F/101 M (48.4)	47–75	Serum LBP	M = F	positive association with arterial stiffness ↑ M	Sakura et al. (2017) [106]
**CHD**	Cross-sectional	British multi-ethnic cohort/ Healthy subjects (N = 192)	96 F/96 M (50)	40–59	Plasma LPS	↑ M ↑ South Asians > White > Africans	positive association with estimated CHD risk	Miller et al. (2009) [75]
**CHD**	Prospective nested case–control	North American male cohort (HPFS)/ Incident CHD cases (N = 496) Controls (N = 496)	0 F/496 M (0)	40–75	Baseline plasma LBP and sCD14	ND	no association with CHD risk positive association with BMI, CRP negative association with HDL-cholesterol	Wang et al. (2025) [108]
**CHD**	Prospective nested case–control	North American female cohort (NHSII)/ Incident CHD cases (N = 212) Controls (N = 212)	212 F/0 M (100)	25–42	Baseline plasma sCD14	ND	positive association with CHD risk association with CRP, total/HDL-cholesterol	Wang et al. (2025) [108]
**Subclinical vascular injury**	Cross-sectional	Italian cohort/ Ob children/adolescents (N = 60)	36 F/24 M (60)	8–18	Serum LPS	↑ M	positive correlation with TNF-a, PAI-1, sICAM-1, MMP-9, MPO, VEGF	Varma et al. (2015) [109]
***Gastrointestinal cancers***								
**Colorectal adenoma**	Clinical trial	American cohort/ 193 subjects with adenomatous polyposis	71 F/122 M (37)	30–74	Serum anti-LPS IgA	↑ M	positive association with BMI	Yang et al. (2016) [114]
**CRC**	Prospective case–control	European cohort (EPIC)/ Incident CRC cases (N = 1065; colon N = 667; rectal N = 398) Controls: 1065	368 F/299 M (55.5, colon) 187 F/211 M (47, rectal)	58.1 (6.8)	Baseline serum anti-LPS IgA/IgG	M = F	positive association with CRC risk ↑ M	Kong et al. (2016) [115]
**CRC**	Nested case–control/	Multi-ethnic cohort/ Incident CRC cases (N = 819) Controls (N = 819)	Cases/Controls: 366 F/453 M (44.7)	45–75	Baseline plasma LBP	M = F	no association with CRC risk M = F	Citronberg et al. (2018) [116]
***Neurodegenerative diseases***								
**PD**	Prospective case–control	European cohort (EPIC)/ Incident PD cases (N = 352) Controls (N = 352)	PD/Controls: 157 F/195 M (45)	PD cases: 54–65 Controls: 55–65	Baseline plasma LBP	in controls M = F ↑ in PD cases ↑ F	association with PD risk ↑ F ↑ Ow/Ob	Zhao et al. (2023) [128]
**Chronic pain**	Randomized controlled	Sweden/ Healthy subjects receiving an intravenous injection of LPS (0.6 ng/kg) or saline (N = 52)	i.v. LPS: 18 F/13 M Saline: 11 F/10 M (55.7)	18–50	ND	ND	i.v. LPS injection: impaired conditioned pain modulation ↑ F Induced plasma IL-6 and IL-8 ↑ F	Karshikoff et al. (2015) [133]
**Chronic pain**	Randomized controlled crossover	German/ Healthy subjects receiving an intravenous injection of LPS (0.4 ng/kg) or saline (N = 40)	20 F/20 M (50)	18–45	ND	ND	i.v. LPS injection: induced state anxiety, visceral and musculoskeletal hyperalgesia M = F induced IL-6, TNF-a ↑ F	Wegner et al. (2015) [44]
***Aging***								
**Aging**	Prospective	North American/ 95 healthy participants: 17 young adults 41 adults 37 older adults	54 F/41 M (56.8)	20–100	Serum LPS	Markers of barrier function ↓ F Age-related increase in LPS, TNF-a/IL-10 ratio, VEGF-A, CRP ↑ F	ND	Quin et al. (2024) [145]

CHD, Coronary heart disease; CRC, colorectal cancer; C-reactive protein; F, females; HDL, high-density lipoproteins; Ig, immunoglobulin; i.v., intravenous; LPS, lipopolysaccharide; LBP, LPS binding protein; MetS, metabolic syndrome; M, males; ND, not determined; Ob, obese: Ow, overweight; PD, Parkinson’s disease; sCD14, soluble CD14; T2DM, type 2 diabetes mellitus; ↑↓ F, higher/lower in females than males; ↑↓ M, higher/lower in males than females.

**Figure 3:**
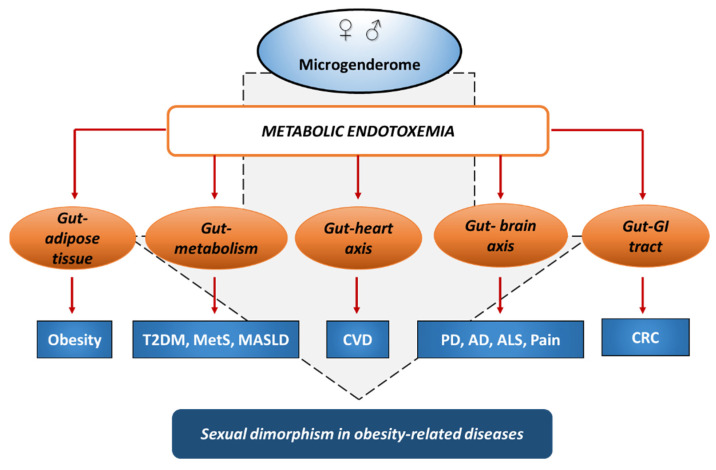
The impact of microgenderome and the potential role of metabolic endotoxemia on obesity-associated diseases. (AD, Alzheimer’s disease; ALS, Amyotrophic Lateral Sclerosis; CRC, colorectal cancer; CVD, cardiovascular diseases; MASLD, metabolic dysfunction-associated steatotic liver disease; MetS, metabolic syndrome; PD, Parkinson’s disease; T2DM, type 2 diabetes mellitus).

Overall, these studies indicate that metabolic endotoxemia is associated with the risk of several diseases, showing sexual dimorphism in onset and progression, and involving gut dysbiosis and gut connections with distal body districts and organs. Sex and gender differences in microbiome and immune system, collectively identified as microgenderome, can influence metabolic endotoxemia and its impact on human health (Figure 3).

## 6. Conclusions

This review highlights the key role of endogenous endotoxins in driving systemic low-grade inflammation and promoting obesity and related chronic diseases by connecting the gut with the involved organs.

Despite the large body of evidence on the link between metabolic endotoxemia and obesity, only a limited number of studies have focused on sex differences. Available data on baseline endotoxemia in both healthy individuals and subjects with obesity show an association of the male sex with higher blood endotoxin levels, although studies on young women with obesity reported opposite results. Notably, controlled endotoxin challenge studies have revealed clear sex differences in immune responses to acute LPS exposure, showing a higher inflammatory response in healthy women. This highlights that the innate response to endotoxins can be influenced by the hormonal status, the experimental conditions, and the nature (acute versus chronic) of the exposure, as well as by gender-related factors. In this regard, although sex-stratified data on postprandial endotoxemia are very limited, the importance of dietary habits in regulating metabolic endotoxemia has been extensively reported, with healthy and unhealthy diets showing opposite effects.

The impact of sex on the connection between endotoxemia and obesity-related NCDs has instead gained increasing attention in recent years. The stronger immune response and faster resolution of inflammation observed in women compared to men can contribute to delaying the metabolic derangements and the onset of cardiometabolic diseases and cancer. In support of this hypothesis, circulating endotoxin levels were found to more frequently associate with the risk of CVD and CRC in men than women, according to the higher incidence or earlier onset of disease in men. On the other hand, in the context of ND, very few studies to date have investigated sex differences, and higher endotoxemia and inflammation in women seem to be associated with increased intensity of chronic pain and a higher risk of PD, according to accelerated inflammaging.

## 7. Future Perspectives

Future research should focus on elucidating the precise molecular mechanisms underlying the complex interplay among genetic, hormonal, and environmental factors in the sex differences observed in metabolic endotoxemia. The consistent inclusion of sex as a biological variable in study design, data analysis, and reporting is crucial for advancing our knowledge on sex disparities in health and disease, and for aiming at personalized prevention and treatment strategies in chronic diseases. Furthermore, studies aimed at a deeper understanding of how specific nutrients or dietary interventions affect metabolic endotoxemia and how the effects of diet on endotoxemia and inflammation are influenced by sex and gender are advisable. The sex differences in the level of endotoxemia and in its modulation by diet could allow the identification of high-risk individuals who would benefit from lifestyle interventions and could help guide prevention towards sex-oriented strategies.

## Data Availability

No new data were created or analyzed in this study. Data sharing does not apply to this article.
